# Prevalence of Polyherbacy in Ambulatory Visits to Traditional Chinese Medicine Clinics in Taiwan

**DOI:** 10.3390/ijerph120809639

**Published:** 2015-08-14

**Authors:** Ming-Hwai Lin, Hsiao-Ting Chang, Chun-Yi Tu, Tzeng-Ji Chen, Shinn-Jang Hwang

**Affiliations:** 1 Department of Family Medicine, Taipei Veterans General Hospital, No. 201, Sec. 2, Shi-Pai Road, Taipei 112, Taiwan; E-Mails: minghwai@gmail.com (M.-H.L.); htstar@gmail.com (H.-T.C.); sjhwang@vghtpe.gov.tw (S.-J.H.); 2 School of Medicine, National Yang-Ming University, No. 155, Sec. 2, Linong Street, Taipei 112, Taiwan; 3 Department of Family Medicine, Taipei Veterans General Hospital, Taoyuan branch, No. 100, Sec. 3, Cheng Kung Road, Tao Yuan 330, Taiwan; E-Mail: cytu1965@gmail.com (C.-Y.T.);

**Keywords:** traditional Chinese medicine, polypharmacy, polyherbacy, drug interaction, complementary and alternative medicine, National Health Insurance

## Abstract

Patients with a polyherbal prescription are more likely to receive duplicate medications and thus suffer from adverse drug reactions. We conducted a population-based retrospective study to examine the items of Chinese herbal medicine (CHM) per prescription in the ambulatory care of traditional Chinese medicine (TCM) in Taiwan. We retrieved complete TCM ambulatory visit datasets for 2010 from the National Health Insurance database in Taiwan. A total of 59,790 patients who received 313,482 CHM prescriptions were analyzed. Drug prescriptions containing more than five drugs were classified as polyherbal prescriptions; 41.6% of patients were given a polyherbal prescription. There were on average 5.2 ± 2.5 CHMs: 2.3 ± 1.1 compound herbal formula items, and 3.0 ± 2.5 single Chinese herb items in a single prescription. Approximately 4.6% of patients were prescribed 10 CHMs or more. Men had a lower odds ratio (OR) among polyherbal prescriptions (OR = 0.96, 95% confidence interval [CI] 0.92–0.99), and middle-aged patients (35–49 years) had the highest frequency of polyherbal prescription (OR = 1.19, 95% CI = 1.13–1.26). Patients with neoplasm, skin and subcutaneous tissue disease, or genitourinary system disease were more likely to have a polyherbal prescription; OR = 2.20 (1.81–2.67), 1.65 (1.50–1.80), and 1.52 (1.40–1.64), respectively. Polyherbal prescription is widespread in TCM in Taiwan. Potential herb interactions and iatrogenic risks associated with polyherbal prescriptions should be monitored.

## 1. Introduction

Polypharmacy has been recognized as a public health problem. Polypharmacy is associated with health-related problems such as adverse drug reactions, drug interactions, and increased risk of hospitalization [1]. Danijela *et al.* suggested the use of five or more medications in the current definition of polypharmacy to estimate the medication-related adverse effects for frailty, disability, mortality, and falls [2]. Nisly *et al.* described the excessive and inappropriate use of herbal supplements, and the term “polyherbacy” was coined [3].

According to the World Health Organization, 75% of the world’s population is using herbs for basic healthcare needs. Many pharmaceutical drugs are derived directly from both natural and traditional remedies distributed around the world [4]. A considerable consequence resulting from polypharmacy or polyherbacy is the potential for drug–herb interactions to occur among various products [5,6]. The use of multiple medications and herbs can lead to adverse consequences, particularly for elderly people and cancer patients [7,8,9]. Patients with polyherbal prescription are more likely to receive duplicate medications and thus suffer from adverse drug reactions [6,10]. Because relevant research in TCM is limited, the current study aims to explore the prevalence and the associated influencing factors of polyherbacy in ambulatory visits to traditional Chinese medicine clinics in Taiwan.

The National Health Insurance (NHI) program in Taiwan, established in 1995, covers almost all residents of Taiwan (99.5% at the end of 2010). Reimbursement for TCM has been included in the program since 1996. Only officially accredited TCM physicians are qualified for reimbursement from the NHI, and they are permitted to practice either in the outpatient departments of hospitals or in clinics. At the end of 2010, more than 2700 TCM clinics were contracted to the Bureau of NHI (BNHI), providing TCM ambulatory care and services such as oral medicine, acupuncture, and manipulative therapy. These qualified institutions obtain reimbursement from the NHI program in accordance with the official fee schedule [11].

Beneficiaries can choose freely between Western medicine practitioners and TCM doctors. Approximately 28.4% of the 23 million residents in Taiwan use the NHI for TCM [12]. The widespread use of TCM in Taiwan is not surprising because TCM has been in use in China for more than 2000 years and the ancestors of most Taiwanese people are immigrants from China since the 17th century. These factors account for the widespread use of TCM in Taiwan [13]. Because all claims data are available in an electronic form, a large-scale survey can be feasibly conducted.

According to the TCM theory, a classical prescription may contain multiple herbal drugs, namely a compound herbal formula (HF), also called a remedy or Fang-Ji. HF is a combination of compatible herbal drugs in fixed dosages ascribed to well-known Chinese textbooks of medicine and is known to reduce the adverse effects of herbs. Licensed TCM doctors prescribe a typical CHM prescription, which contains Chinese HFs and/or Chinese herbs (CHs), to achieve additive or synergistic effects after examination of the medical history and physical evaluation of a patient [14].

Yi found that the commercial products of herbal extract powders, including different types of HFs and/or CHs in a prescription, and traditional Chinese HFs contain 1–13 herbs [15]. In this study, we investigated the prevalence of polypharmacy in TCM ambulatory visits in Taiwan and identified patients with a polyherbal prescription by analyzing the NHI claims data of 2010.

## 2. Methods

### 2.1. Data Source

The NHI program was initiated in Taiwan in 1995 and covered all 23,074,487 beneficiaries (nearly all the inhabitants of Taiwan) at the end of 2010, which is equivalent to a coverage rate of 99.5%. In 1999, the BNHI released all claims data in an electronic format to the public under the NHI Research Database (NHIRD) project. The structure of the claims files is described in detail on the NHIRD website [16] and in previous studies [4,5].

We obtained the complete TCM ambulatory visit datasets of 2010 (CM_CD2010.DAT and CM_OO2010.DAT) from the NHIRD, including the office visit files and corresponding patient profiles for 2010 in Taiwan. The visit files, CM_CD2010.DAT, contained the dates of encounters, medical care facilities, genders, dates of birth, major diagnoses, and medical costs. Although the concept of disease entities in TCM is considerably different from that in Western medicine, TCM physicians are requested to code office visit claims with a diagnosis based on the International Classification of Diseases, Ninth Revision, Clinical Modification (ICD-9-CM) [11,12] with no more than three diagnostic codes at each visit when claiming reimbursement.

For privacy protection, the unique identifiers of the patients and institutions were scrambled cryptographically to ensure anonymity.

### 2.2. Study Design

To analyze the CHM prescriptions in the population, we constructed a systematic sampling of the TCM data subsets. Two thousandths (0.2%) of the ambulatory care expenditures, by visit, was extracted by systematic sampling method on a monthly basis, together with the related records in details of ambulatory care orders. The prescription files, CM_OO2010.DAT, contained corresponding prescriptions of Chinese herbal drugs or formulas. The CHs or HFs are prepared as a concentrated powder or fine granules in Taiwan. A prescription may contain one or more CHs or HFs. These can be easily mixed and dispensed into small packages to take one prescription at a time.

We then performed statistical analyses on the number of CHMs (CHs or HFs) per prescription and the duration of these prescriptions. Moreover, we identified the most commonly prescribed CHs and HFs.

### 2.3. Statistical Analysis

We used Microsoft SQL Server 2008 (Microsoft Corp., Redmond, WA, USA) for data linkage analysis and processing. Descriptive data, including the mean (standard deviation) and frequencies (percentage), were presented as continuous variables and discrete variables, respectively. The anatomical therapeutic classification was applied for coding the prescription drugs.

SPSS for Windows Version 18.0 (SPSS, Inc., Chicago, USA) was used for data management and statistical analysis. Multivariate logistic regression was used to estimate the odds ratio (OR) as a measure of association of CHM polyherbal prescription. OR estimates and 95% confidence intervals (CIs) were calculated for different levels of significant variables and not each variable’s corresponding reference level. A *p* value of less than 0.05 was considered statistically significant.

This study was exempted from review by the Institutional Review Board because of the NHIRD’s deidentified nature.

## 3. Results

Among the 23,074,487 valid beneficiaries of the NHI at the end of 2010 in Taiwan, 6,704,367 (29.1%) had used TCM. The total TCM services in 2010 reached 36,726,496, and the average number of visits for TCM services of a patient was 5.5 (female = 5.8, male = 5.0); 5,281,991 (84.9%) of them had been prescribed CHM drugs in 2010.

After two thousandths (0.2%) systematic sampling, 59,790 patients who received a total of 313,482 CHM items (single herb or compound formula) were analyzed. Of the 313,482 CHMs prescribed, 177,876 (56.7%) were single CHs and 135,606 (43.2%) were Chinese HFs. Most commonly, the prescriptions of CHM in Taiwan contained five drugs (17.7%) in a single prescription, followed by four (16.4%), and six (15.6%) (Table 1). There was an average of 5.2 ± 2.5 CHM items: 2.3 ± 1.1 compound HFs and 3.0 ± 2.5 CHs in a single prescription.

**Figure 1:**
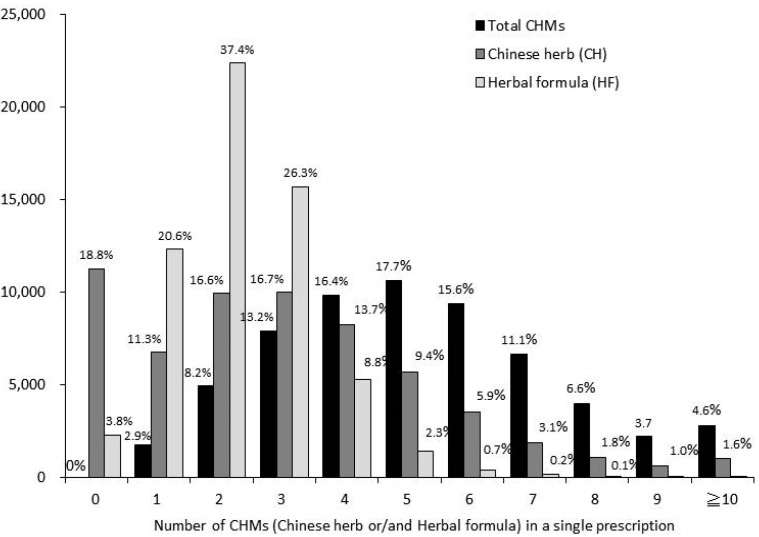
Number of CHMs (Chinese herbs and/or herbal formulas) in a single prescription.

Approximately 37.4% CHM prescriptions contained two HFs in a single prescription, followed by three (26.3%), and one (20.6%) (Figure 1). The average item of HFs prescribed was 2.3 ± 1.1. Approximately 18.8% of CHM prescription contained one CH herb in a single prescription, followed by four (16.7%), and three (16.6%). The average item of CHs prescribed was 3.0 ± 2.5. (Figure 1).

**Table 1:** Number of Chinese herbal medicine (CHM) drugs per prescription.

Number of CHM	1	2	3	4	5	6	7	8	9	≧10
Count	1713	4906	7883	9832	10594	9336	6608	3960	2182	2776
Percentage	2.9	8.2	13.2	16.4	17.7	15.6	11.1	6.6	3.7	4.6
Cum. Percentage	2.9	11.1	24.3	40.7	58.4	74	85.1	91.7	95.4	100
No. of CH	0	1	2	3	4	5	6	7	≧8	
Count	11259	6733	9908	9980	8194	5642	3525	1875	2674	
Percentage	18.8	11.3	16.6	16.7	13.7	9.4	5.9	3.1	3.5	
Cum. Percentage	18.8	30.1	46.7	63.4	77.1	86.5	92.4	95.5	100	
No. of HF	0	1	2	3	4	5	≧6			
Count	2261	12294	22347	15693	5251	1381	563			
Percentage	3.8	20.6	37.4	26.3	8.8	2.3	0.9			
Cum. Percentage	3.8	24.3	61.7	88.0	96.8	99.1	100			

S.D.: standard deviation.

Number of prescription

More than half of patients (58.4%) received 1–5 CHMs per prescription, and 4.6% of the patients were prescribed 10 CHMs or more (Table 1). The most common duration of a prescription was 7 days (47.9%), followed by less than 7 days (44.6%), and more than 7 days (7.5%).

Among the 439 CHs, the most frequently prescribed CH was Yan-hu-suo (5.9%), followed by Da-huang (5.8%), Jie-geng (5.4%), Huang-qin (5.1%), and Gan-cao (5.0%) (Table 2). The 100 most commonly prescribed CHs cover more than 70% of all prescriptions, and 188 types of CHs encompass more than 90% of all prescriptions.

**Table 2:** Top 20 single Chinese herbs (CHs) prescribed in Taiwan in 2010 (total prescription number, *n* = 59,790).

	Chinese Name	Single Chinese Herb	Generic Name	Number of Prescription	Percentage
1	延胡索	Yan-hu-suo	Rhizoma Corydalis	3539	5.9%
2	大黃	Da-huang	Radix et Rhizoma Rhei	3469	5.8%
3	桔梗	Jie-geng	Radix Platycodi	3209	5.4%
4	黃芩	Huang-qin	Radix Scutellariae	3047	5.1%
5	甘草	Gan-cao	Radix Glycyrrhizae	2974	5.0%
6	貝母	Bei-mu	Bulbus Fritillariae Thunbergii	2884	4.8%
7	丹參	Dan-shen	Radix Salviae Miltiorrhizae	2541	4.3%
8	白芷	Bai-zhi	Radix Angelicae Dahuricae	2445	4.1%
9	葛根	Ge-gen	Radix Puerariae	2435	4.1%
10	黃耆	Huang-qi	Radix Astragali seu Hedysari	1963	3.3%
11	厚朴	Hou-pu	Cortex Magnoliae Officinalis	1927	3.2%
12	杏仁	Xing-ren	Semen Armeniacae Amarum	1919	3.2%
13	香附	Xiang-fu	Rhizoma Cyperi	1899	3.2%
14	麥門冬	Mai-men-dong	Radix Ophiopogonis	1879	3.1%
15	玄參	Xuan-shen	Radix Scrophulariae	1794	3.0%
16	連翹	Lian-qiao	Fructus Forsythiae	1776	3.0%
17	海螵蛸	Hai-piao-xiao	Endoconcha Sepiae	1753	2.9%
18	魚腥草	Yu-xing-cao	Herba Houttuyniae	1753	2.9%
19	杜仲	Du-zhong	Cortex Eucommiae	1630	2.7%
20	夜交藤	Ye-jiao-teng	Caulis Polygoni Multiflori	1627	2.7%

Among the 303 Chinese HFs, the most frequently prescribed HF was Jia-wei-xiao-yao-san (7.8%), followed by Ge-geng-tang (5.0%), Xin-yi-qing-fei-tang (5.0%), Shu-jing-huo-xue-tang (4.5%), and Ban-xia-xie-xin-tang (4.3%) (Table 3). The 100 most commonly prescribed HFs cover 79.4% of all prescriptions, and 145 types of HFs encompass more than 90% of all prescriptions. “Formulas for relieving superficial syndrome” was the most commonly prescribed HF (23.6%), followed by “formulas for harmonizing” (21.6%) and “formulas for tonifying” (20.4%) (Table 4).

**Table 3:** Top 20 Chinese herbal formulas (HFs) prescribed in Taiwan in 2010 (total prescription number, *n* = 59,790).

Chinese Herbal Formulas (Chinese Name)		Number of Ingredient	Classification and Therapeutic effect	Number of Prescription (%)	
1	Jia-wei-xiao-yao-san	10	**Harmonizing liver and spleen** It may relieve symptoms such as anxiety, irritability, insomnia, and depression due to life stress, premenstrual tension, or postmenopausal syndrome.	4679	7.8
2	Ge-geng-tang	7	**Relieving superficial syndrome** It may relieve symptoms of common cold and headache caused by external wind and cold.	2989	5.0
3	Xin-yi-qing-fei-tang	9	**Relieving superficial syndrome** It may relieve symptoms of chronic rhinitis, nasal congestion, or chronic cough caused by lung heat, and disseminate Lung Qi.	2976	5.0
4	Shu-jing-huo-xue-tang	17	**Regulating blood (improve blood circulation)** It may relieve symptoms of arthritis, numbness, gout, sciatica, and lumbago via relaxing the channels and invigorating the blood decoction.	2686	4.5
5	Ban-xia-xie-xin-tang	7	**Harmonizing stomach and spleen** It may relieve symptoms of gastroenteritis, hepatitis, ulcer, cholecystitis, liver cirrhosis, coronary artery disease, and chronic fatigue caused by disharmony between the stomach and intestines.	2584	4.3
6	Shao-yao-gan-cao-tang	2	**Formulas for harmonizing** It may relieve symptoms of sciatica, toothache, trigeminal neuralgia, and hernia pain caused by disharmony between the liver and spleen.	2518	4.2
7	Chuan-xiong-cha-tiao-san	8	**Expelling external wind** It may relieve headache and stuffy nose due to wind attack via dispelling wind.	2427	4.1
8	Ping-wei-san	6	**Eliminating dampness** It may relieve symptoms of gastroenteritis, intestinal obstruction, coronary artery disease, and peptic ulcer.	2409	4.0
9	Yin-qiao-san	10	**Relieving superficial syndrome with pungent and cool** It may relieve symptoms of common cold, influenza, sinusitus, and herpes simplex.	2405	4.0
10	Xiao-qing-long-tang	7	**Relieving superficial syndrome with pungency and warmth** It may relieve symptoms of bronchitis, allergic rhinitis, emphysema, and sinusitis.	2236	3.7
11	Ma-xing-gan-shi-tang	4	**Relieving superficial syndrome with pungency and cold** It may relieve symptoms of acute tracheitis, lobar pneumonia, fever, and bronchial asthma caused by lung heat, and disseminate Lung Qi.	2220	3.7
12	Ma-zi-ren-wan	6	**Purging with moistening/lubricating herbs** It may relieve symptoms of constipation, hemorrhoids, chronic colitis, and gastritis.	2080	3.5
13	Xin-yi-san	9	**Relieving superficial syndrome** It may relieve symptoms of nasal polyps, sinus congestion, the common cold, and allergic rhinitis.	2042	3.4
14	Xiao-chai-hu-tang	7	**Harmonizing Shao–yang** It may relieve symptoms of influenza, upper respiratory tract infection, bronchial asthma, and gastritis.	2035	3.4
15	Cang-er-san	4	**Expelling external Wind** It may relieve symptoms of acute and chronic rhinitis, acute and chronic sinusitis and allergic rhinitis.	1911	3.2
16	Gan-lu-yin	10	**Treating dryness diseases** It may relieve symptoms of blepharitis, gingivitis, conjunctivitis, and jaundice.	1908	3.2
17	Xiang-sha-liu-jun-zi-tang	10	**Improving digestion** It may relieve symptoms of indigestion, gastritis, bronchitis, and ulcers.	1874	3.1
18	Suan-zao-ren-tang	5	**Tranquilizing the mind with nourishing** It may relieve symptoms of insomnia, nightmares, amnesia, and premenopause syndrome.	1746	2.9
19	long-dan-xie-gan-tang	9	**Clearing heat in the Zang-fu (organ network)** It may relieve symptoms of psoriasis, migraine, eczema, and eye problems.	1649	2.8
20	Liu-wei-di-huang-wan	6	**Tonifying (Yin tonic)** It may relieve symptoms of menopause, hypertension, Addison’s disease, and coronary heart disease.	1629	2.7

**Table 4:** The frequency of Chinese herbal formulas (HFs) prescribed by classifications in Taiwan in 2010 (total prescription number *n* = 59,790).

Drug Type	Classification of Formulas	Number of Prescription	(%)
發表之劑	**Formulas for relieving superficial syndrome** These formulas treat disharmonies in the superficial portion of the body through the actions of inducing sweating, releasing muscles, or promoting elimination. The indications of these include dislike of cold, fever, headache, pain in the body, and headache.	14,080	23.6
和解之劑	**Formulas for harmonizing** These formulas are useful for a range of psychological or emotion-related imbalance conditions. The indications of these formulas include bronchitis, hepatitis, hypertension, anxiety, depression, dysmenorrhea, and more along with a range of digestive problems.	12,919	21.6
補養之劑	**Formulas for tonifying** These formulas enrich, nourish, or replenish the qi, blood, yin, and yang of the body when they are deficient or weak. The indications of these formulas range include pale face, weak voice, shortness of breath and fatigue, poor appetite and energy, senile lower energy or lower energy after sickness, cold limbs, impotence, loose stool, urinary incontinence, and infertility.	12,202	20.4
清熱瀉火劑	**Formulas for clearing heat** These formulas treat various heat syndromes through the actions of clearing away heat, draining fire, cooling blood, eliminating the toxicity, and nourishing the body. Typical heat signs include high fever, profuse sweating, a surging pulse, excessive thirst, bleeding, and constipation. The formulas are often used for relatively short periods with somewhat acute conditions.	12,113	20.3
表裡之劑	**Formulas for relieving interior/exterior** These formulas mediate or regulate the physiological functions needed so as to achieve a new balanced condition. They are usually used in Shao Yang diseases, disharmonies between the liver and spleen, and gastrointestinal problems.	8378	14.0
理血之劑	**Formulas for regulating blood** This category of formulas treats disorders related to blood flow and involves relieving blood stasis and arresting bleeding. The indications of these formulas includes bleeding problems like epistaxis, bloody stools, hematuria, hypermenorrhea, and blood stasis-related symptoms such as purplish tongue with blood spots.	8327	13.9
潤燥之劑	**Formulas for treating dryness diseases** These formulas treat fluid loss symptoms such as dry, wrinkled, or withered skin, dry hair and scalp, dry mouth and cracked lips, and dry and hard stools.	7718	12.9
袪痰之劑	**Formulas for eliminating phlegm** These formulas aim at improving the functions of the spleen, kidneys, lungs, and liver so as to prevent transformation of internal phlegm. They are useful for a range of disorders, such as coughing, wheezing, nausea, dizziness or vertigo, nodules or lumps, and seizures.	6999	11.7
消導之劑	**Formulas for improving digestion** These formulas promote digestion and remove food retention. They relieve indigestion caused by over­consumption of meat and greasy foods.	6335	10.6
袪風之劑	**Formulas for treating wind-related diseases** Wind evil is considered a yang pathogen and it has influential effects. These formulas relieve wind-related liver and kidney dysfunctions, such as dizziness, vertigo, tremors, convulsions, loss of muscle tone, slurred speech, and sudden loss of consciousness, facial distortion, and paralysis.	5688	9.5
安神之劑	**Formulas for tranquilization** These formulas relieve mental tension and uneasiness and are similar to tranquilizers in Western medicine. The indications of these formulas include anxiety, forgetfulness, disorientation and insomnia, manic behavior, bad temper, and agitation.	5677	9.5
利濕之劑	**Formulas for eliminating dampness** Disturbances in water metabolism can lead to dampness disorders that cause edema, urinary difficulty, tiredness, heavy limbs, stiffness and pain in the joints, and respiratory symptoms. These formulas dispel dampness through drying, excreting, facilitating urination, and purgation.	4440	7.4
袪寒之劑	**Formulas for warming the interior** These formulas warm the interior and unblock the meridians to eliminate cold substances inside the body. The indications of these formulas include chills, fatigue, gastric discomfort, increased urine output, loose bowels, and cold extremities.	3340	5.6
經產之劑	**Formulas for women’s diseases** These formulas are indicated for problems in pregnant women and breast-feeding women.	3018	5.1
理氣之劑	**Formulas for regulating Qi** Qi stagnation is characterized by fullness, pain that is accompanied by a distended sensation, and a preference for belching or breaking wind. These formulas are usually for treating problems like vomiting, hiccups, belching, and some forms of coughing or wheezing.	2621	4.4
攻裡之劑	**Formulas for purgation** These formulas break down interior accumulations through vigorous evacuation of the bowels. They are used as laxatives for relieving intestinal stagnancy and removing heat, fire, toxins, and all retained fluids.	2246	3.8
清暑之劑	**Formulas for clearing heat in Qi portion/level** Clears Qi-level heat, drains stomach fire, generates fluids, and relieves thirst.	2172	3.6
癰瘍之劑	Formulas for abscess These formulas can treat all types of boils and carbuncles with localized erythema, swelling, heat and pain with fever chills, red tongue with yellow coating, and rapid pulse.	2155	3.6
收濇之劑	Formulas for astringency These formulas prevent abnormal discharge or leakage of fluids and other substances from the body, such as sweat, sputum, blood, urine, stool, sperm, and vaginal discharges.	1051	1.8

Table 5 and Table 6 list the most commonly used HF combinations. The most common combination of two HFs was Shao-yao-gan-cao-tang plus Shu-jing-huo-xue-tang (0.88%), and the most common combination of three HFs was Shao-yao-gan-cao-tang, Shu-jing-huo-xue-tang, and Du-huo-ji-sheng-tang (0.10%). Both combinations are formulas for relieving neuromuscular pain. We summed up the ingredients of different CHM combinations; according to the results, the herb items ranged from 8 to 32 and from 17 to 46 when two-formula and three-formula combinations were used.

**Table 5:** The most common two-formula combinations of Chinese herbal medicine (CHM) in a single prescription in Taiwan in 2010 (total prescription number *n* = 59,790).

	Chinese Herbal Formulas (Chinese Name)	Number of Ingredients	Number of Prescriptions (%)	
1	Shao-yao-gan-cao-tang + Shu-jing-huo-xue-tang	19	529	0.88
2	Shu-jing-huo-xue-tang + Du-huo-ji-sheng-tang	32	388	0.65
3	Jia-wei-xiao-yao-san + Suan-zao-ren-tang	15	373	0.62
4	Xiao-qing-long-tang + Xin-yi-san	16	366	0.61
5	Ban-xia-xie-xin-tang + Ping-wei-san	13	335	0.56
6	Xin-yi-qing-fei-tang + Yin-qiao-san	19	329	0.55
7	Ma-xing-gan-shi-tang + Yin-qiao-san	14	327	0.55
8	Chuan-xiong-cha-tiao-san + Ge-geng-tang	15	324	0.54
9	Xin-yi-qing-fei-tang + Cang-er-san	13	284	0.47
10	Xin-yi-qing-fei-tang + Ma-xing-gan-shi-tang	13	268	0.45
11	Jia-wei-xiao-yao-san + Gan-mai-da-zao-tang	13	254	0.42
12	Xiao-qing-long-tang + Xin-yi-qing-fei-tang	16	246	0.41
13	Ban-xia-xie-xin-tang + An-zhong-san	14	227	0.38
14	Tian-wang-bu-xin-dan + Jia-wei-xiao-yao-san	23	222	0.37
15	Tian-wang-bu-xin-dan + Suan-zao-ren-tang	18	214	0.36
16	Shu-jing-huo-xue-tang + Dang-gui-nian-tong-tang	31	211	0.35
17	Jia-wei-xiao-yao-san + Dang-gui-shao-yao-san	16	209	0.35
18	Shao-yao-gan-cao-tang + Du-huo-ji-sheng-tang	17	208	0.35
19	Ma-xing-gan-shi-tang + Cang-er-san	8	206	0.34
20	Xiao-qing-long-tang + Cang-er-san	11	204	0.34

**Table 6:** The most common three-formula combination of Chinese herbal medicine (CHM) in a single prescription in Taiwan in 2010 (total prescription number *n* = 59,790).

	Chinese Herbal Formulas (Chinese Name)	Number of Ingredients	Number of Prescriptions (%)		
1	Shao-yao-gan-cao-tang + Shu-jing-huo-xue-tang + Du-huo-ji-sheng-tang	34	62	0.10
2	Chuan-xiong-cha-tiao-san + Gan-lu-yin + Xing-su-yin	29	57	0.10
3	Chuan-xiong-cha-tiao-san + Liu-he-tang + Gan-lu-yin	31	56	0.09
4	Xin-yi-qing-fei-tang + Ma-xing-gan-shi-tang + Yin-qiao-san	23	49	0.08
5	Ba-zheng-san + Wu-lin-san + Di-dang-tang	17	47	0.08
6	Shao-yao-gan-cao-tang + Shu-jing-huo-xue-tang + Dang-gui-nian-tong-tang	33	45	0.08
7	Xiao-qing-long-tang + Xin-yi-san + Xiang-sha-liu-jun-zi-tang	26	44	0.07
8	Chuan-xiong-cha-tiao-san + Gan-lu-yin + Shu-jing-huo-xue-tang	35	43	0.07
9	Chuan-xiong-cha-tiao-san + Gan-lu-yin + Xin-yi-san	27	41	0.07
10	Chuan-xiong-cha-tiao-san + Gan-lu-yin + Sheng-mai-yin	21	38	0.06
11	Jia-wei-xiao-yao-san + Gan-mai-da-zao-tang + Suan-zao-ren-tang	18	38	0.06
12	Chuan-xiong-cha-tiao-san + Xing-su-yin + Xin-yi-san	28	37	0.06
13	Gan-lu-yin + Xing-su-yin + Xin-yi-san	30	37	0.06
14	Shao-yao-gan-cao-tang + Shu-jing-huo-xue-tang + Ge-geng-tang	26	37	0.06
15	Ma-xing-gan-shi-tang + Cang-er-san + Yin-qiao-san	18	36	0.06
16	Tian-wang-bu-xin-dan + Jia-wei-xiao-yao-san + Suan-zao-ren-tang	28	36	0.06
17	Shu-jing-huo-xue-tang + Dang-gui-nian-tong-tang + Du-huo-ji-sheng-tang	46	33	0.06
18	Jia-wei-xiao-yao-san + Chai-hu-jia-long-gu-mu-li-tang + Suan-zao-ren-tang	27	32	0.05
19	Tian-wang-bu-xin-dan + Chai-hu-jia-long-gu-mu-li-tang + Suan-zao-ren-tang	30	32	0.05
20	Shao-yao-gan-cao-tang + Shen-tong-zhu-yu-tang + Shu-jing-huo-xue-tang	31	31	0.05

A total of 24,862 patients (41.6%) had prescriptions containing more than five CHM drugs; such prescriptions were classified as polyherbal prescriptions. Table 7 shows the comparison of patient profiles between polyherbal prescriptions and other prescriptions. Male patients had a lower frequency of polyherbal prescription than did female patients (40.4% *vs.* 42.3%, *p* < 0.001), and middle-aged patients (35–49 years) had the highest frequency of polyherbal prescription (43.3%, *p* < 0.001). Patients whose CHM prescription lasted for more than 7 days had the highest frequency of polyherbal prescription (43.5% *vs.* 39.1%, *p* < 0.001).

Table 8 shows the factors associated with the polyherbal prescription of CHM in a multivariate logistic regression. Men had lower ORs of polyherbal prescription than did women (OR = 0.96, 95% CI = 0.92–0.99), and middle-aged patients (35–49 years) had a higher frequency of polyherbal prescription than did younger patients (<20 years) (OR = 1.19, 95% CI = 1.13–1.26). Patients with a long duration of prescription (≥7 days) were commonly given a polyherbal prescription (OR = 1.67, 95% CI = 1.57–1.78). Furthermore, patients with neoplasm (Ch. 2), skin and subcutaneous tissue disease (Ch. 12), and genitourinary system disease (Ch. 10) more likely had a polyherbal prescription compared with patients with musculoskeletal system and connective tissue diseases (Ch. 13); OR = 2.20 (1.81–2.67), 1.65 (1.50–1.80), and 1.52 (1.40–1.64), respectively.

**Table 7:** Characteristics of patients taking ≤5 and >5 Chinese herbal medicine (CHM) drugs in a single prescription in 2010.

Patient Characteristics		Patients No. (%) of		
		All Visits (*n* = 59,790)	Drug Items ≤5 (*n* = 34,928, 58.4%)	Drug Items >5 (*n* = 24,862, 41.6%)
Sex			*p* < 0.001	
	Male	21,869 (36.6)	13,027 (59.6)	8842 (40.4)
	Female	37,921 (63.4)	21,901 (57.8)	16,020 (42.3)
Age (years)			*p* < 0.001	
	<20	8899 (14.9)	5382 (60.5)	3517 (39.5)
	20–34	14,088 (23.6)	8190 (58.1)	5898 (41.9)
	35–49	17,358 (29.0)	9838 (56.7)	7520 (43.3)
	50–64	12,838 (21.5)	7617 (59.3)	5221 (40.7)
	≥60	6607 (11.0)	3901 (59.0)	2706 (41.0)
Drug prescription day			*p* < 0.001	
	<7	26,639 (44.6)	16,205 (60.8)	10,434 (39.1)
	≥7	33,151 (55.4)	18,723 (56.5)	14,428 (43.5)
Diagnostic Grouping (according to ICD-9-CM)				
Ch01	Infectious and Parasitic Diseases	250 ( 0.4)	155 (62.0)	95 (38.0)
Ch02	Neoplasms	464 ( 0.8)	195 (42.0)	269 (58.0)
Ch03	Endocrine, Nutritional, and Metabolic Diseases	998 ( 1.7)	539 (54.0)	459 (46.0)
Ch05	Mental Disorders	552 ( 0.9)	333 (60.3)	219 (39.7)
Ch06	Nervous System and Sense Organs	1905 ( 3.2)	1100 (57.7)	805 (42.3)
Ch07	Circulatory System	1274 ( 2.1)	690 (54.2)	584 (45.8)
Ch08	Respiratory System	12,889 (21.6)	7495 (58.2)	5394 (41.9)
Ch09	Digestive System	9076 (15.2)	5351 (59.0)	3725 (41.0)
Ch10	Genitourinary System	6068 (10.1)	3352 (55.2)	2716 (44.8)
Ch12	Skin and Subcutaneous Tissue	3212 ( 5.4)	1737 (54.1)	1475 (45.9)
Ch13	Musculoskeletal System and Connective Tissue	5229 ( 8.7)	3425 (65.5)	1804 (34.5)
Ch16	Symptoms, Signs and Ill-defined	15,369 (25.7)	8915 (58.0)	6454 (42.0)
Ch17	Injury and Poisoning	2136 ( 3.6)	1453 (68.0)	683 (32.0)
	The Others	368 ( 0.6)	188 (51.1)	180 (48.9)

We omitted Ch. 4 “Diseases of the Blood and Blood-Forming Organs” Ch. 11 “Complications of Pregnancy, Childbirth, and the Puerperium” Ch. 14 “Congenital Anomalies” and Ch. 15 “Certain Conditions Originating in the Perinatal Period”, because few patients were classified in these groupings.

**Table 8:** Factors associated with the polyherbal prescription (>5 drugs in a single prescription) of Chinese herbal medicine (CHM) in a multivariate logistic regression.

	Univariate Analysis		Multivariable Analysis	
	OR (95% CI)	*p* Value	OR (95% CI)	*p* Value
Sex				
Female	1		1	
Male	0.93 (0.90–0.96)	<0.001	0.96 (0.92–0.99)	0.010
Age (years)				
<20	1		1	
20–34	1.10 (1.04–1.16)	<0.001	1.11 (1.05–1.17)	<0.001
35–49	1.17 (1.11–1.23)	<0.001	1.19 (1.13–1.26)	<0.001
50–64	1.05 (0.99–1.11)	0.090	1.08 (1.02–1.14)	0.009
≧60	1.06 (1.00–1.13)	0.071	1.09 (1.02–1.17)	0.011
drug/day				
<7	1		1	
≧7	1.67 (1.57–1.78)	<0.001	1.67 (1.57–1.78)	<0.001
Diagnosis				
Ch. 13	1		1	
Ch. 1	1.16 (0.90–1.51)	0.256	1.19 (0.92–1.55)	0.190
Ch. 2	2.62 (2.16–3.18)	<0.001	2.20 (1.81–2.67)	<0.001
Ch. 3	1.62 (1.41–1.85)	<0.001	1.41 (1.23–1.62)	<0.001
Ch. 5	1.25 (1.04–1.50)	0.016	1.14 (0.95–1.37)	0.152
Ch. 6	1.39 (1.25–1.55)	<0.001	1.31 (1.17–1.46)	<0.001
Ch. 7	1.61 (1.42–1.82)	<0.001	1.40 (1.23–1.59)	<0.001
Ch. 8	1.37 (1.28–1.46)	<0.001	1.40 (1.31–1.50)	<0.001
Ch. 9	1.32 (1.23–1.42)	<0.001	1.32 (1.23–1.42)	<0.001
Ch. 10	1.54 (1.43–1.66)	<0.001	1.52 (1.40–1.64)	<0.001
Ch. 12	1.61 (1.47–1.76)	<0.001	1.65 (1.50–1.80)	<0.001
Ch. 16	1.37 (1.29–1.47)	<0.001	1.43 (1.34–1.53)	<0.001
Ch. 17	0.89 (0.80–0.99)	0.038	0.90 (0.81–1.00)	0.057

OR: odds ratio; CI: confidence interval; we omitted Ch. 4 “Diseases of the Blood and Blood-Forming Organs” Ch. 11 “Complications of Pregnancy, Childbirth and the Puerperium” Ch. 14 “Congenital Anomalies” and Ch. 15 “Certain Conditions Originating in the Perinatal Period” because few patients were classified in these groupings.

## 4. Discussion

This is the first study to provide a detailed description of polyherbal prescription among TCM patients in the population of Taiwan. This large-scale study on TCM use was feasible only with the aid of a computerized insurance reimbursement database and the progress of a data mining technique.

If we apply the definition of polypharmacy as more than five drugs (not inclusive), 41.6% of the CHM prescriptions are polyherbal prescriptions. The coprescription of three to five CHs or HFs in a single prescription is a common clinical practice in TCM. According to the theory of Chinese medicine, TCM doctors use one or two HFs as the major component to produce main therapeutic actions and the other HFs or CHs for modulating effects, such as improving the pharmacokinetic properties and moderating the harshness, toxicity, and treatment of different symptoms and signs a patient manifests [8].

In 2004, Yi and Chang reported that 92% of the total 11,810 traditional Chinese HFs contained 1–13 herbs [7]. In this study, we identified the most prevalent CHM prescriptions, not only herbs but also different HF combinations. We found that 145 types of HFs and 188 types of CHs encompassed more than 90% of all prescriptions. The most commonly prescribed CMHs for different categorized symptoms are not only valuable for teaching and training about TCM but also helpful in developing new prescription combinations for establishing CHM systematization.

The use of CAM or TCM in Western countries is usually not covered by insurance. Because TCM is reimbursed by the NHI in Taiwan, our study was less affected by the socioeconomic status of the patients [1]. In this study, multivariate analysis showed a significant difference between patients with polyherbal prescription and the other patients without polyherbal prescription with regard to sex, age, prescription duration, and medical diagnosis. The ORs of male patients for polyherbal prescription were lower than those of female patients.

Wang studied the polyherbal prescription behavior for treating upper respiratory tract infection in Taiwan. The results showed a reverse U-shaped relationship between the polyherbal prescription behavior and patient age (the behavior was the strongest in the age range of 18–34 years) [16]. Our result showed that patients aged 35–49 years had the highest OR for polyherbal prescription. Ch. 12, Ch. 14, Ch. 10, and Ch. 11 diseases had a higher OR than did Ch. 13 diseases. These results revealed that the risk factors for polyherbal prescription are strongly associated with clinical diagnosis.

One of the main principles of TCM is the Zang–Xiang theory [17]. The TCM uses eight principles to make different diagnoses, including the manifestations of Yin and Yang syndromes; cold and heat syndromes; deficiency and excess syndromes; and exterior, interior, and half exterior–interior syndromes. The TCM doctors then apply and match and combine CHMs using the TCM theory.

Because the therapeutic effects of HF prescriptions are different or even contrasting for different syndromes, paying attention to syndrome differentiation is crucial. Although CHM is generally safe when used properly, many herbs and formulas have contraindications, and some of them can be toxic if misused. We should pay attention to the toxicity, contraindications, and drug interactions in TCM herbology.

Drug interaction is one of the most crucial concerns in drug safety. Because TCM has become popular, these herbs are often coadministered with therapeutic drugs, thus increasing the possibility of drug–herb interactions, which may have critical clinical significance based on the increasing number of clinical reports of such interactions [10,13]. Moreover, only a few patients disclose their herbal supplement usage to health care providers, and many physicians are unaware of the potential herb–drug interactions.

Numerous studies have reported on reactions between aspirin and CHMs. The interaction between herbs and drugs with narrow therapeutic indices (e.g., warfarin) is a significant safety concern. According to our results, the herb items ranged from 8 to 32 and from 17 to 46 when two-formula and three-formula combinations were used. Approximately 12% of prescriptions contained more than four HFs, which may constitute more than 30 types of CHs. When the combinations of CHM are complex, it is difficult for a TCM practitioner to avoid prescription repetition and recognize herb–drug interaction.

Wang and Jing suggested constructing a system of prescribing a TCM syndrome-corresponding formula to establish a normalized standard in treating diseases effectively and ensuring drug safety. Syndrome-corresponding formula prescription was used, and compound prescription was monitored [14]. Previous studies have shown that prescription drug-monitoring programs reduce the diversity in polyherbal prescriptions [15]. Health care providers can reduce the side effects of polyherbal prescription by using an electronic database.

This study had some limitations. By using datasets from the NHIRD, only officially recognized TCM treatments that were reimbursed under the NHI program in Taiwan could be studied. Thus, the results did not reflect all usage of TCM. People in Taiwan can buy herbal remedies from TCM pharmacies without visiting TCM physicians; such out-of-pocket use of TCM herbs was not recorded. If we include such self-paid TCM treatments, which are not included in the reimbursement data, the frequency would possibly increase. Due to dataset limitations (only the complete records of TCM visits are available for analysis), we focus only on patients with CHM prescriptions. We did not make a direct comparison between traditional Chinese medicine and Western medicine users to see if there are certain pathologies that require consumption of a higher number of medicines. In secondary data analysis, it is difficult to distinguish whether these herbs are consumed because of the side effects of therapeutic medicines or the pathology of the patient. Thus, we should interpret the results cautiously. In the future, studies should consider investigating the underlying factors of polyherbal prescription and the effect on the depletion of health care resources for health policy makers to establish an effective health delivery system.

## 5. Conclusions

In conclusion, this study provided the prescription patterns of CHM. The ingredients, mechanisms, and therapeutic effects of CHs or HFs used to treat diseases must be elucidated and explored in depth. TCM doctors should be aware of potential herb–drug interactions and iatrogenic health risks associated with polyherbal prescriptions.

## Abbreviations

TCM: traditional Chinese medicine
CHM: Chinese herbal medicine
CH: Chinese herb
HF: Herbal formula
CAM: complementary and alternative medicine
NHI: National Health Insurance
NHIRD: National Health Insurance Research Database
BNHI: Bureau of National Health Insurance
ICD-9-CM: International Classification of Diseases, Ninth Revision, Clinical Modification
